# Self‐reported cultural competence among Czech and Slovakian nurses: A comparative correlation study

**DOI:** 10.1111/inr.12969

**Published:** 2024-04-23

**Authors:** Martin Červený, Valérie Tóthová

**Affiliations:** ^1^ Faculty of Health and Social Sciences University of South Bohemia in Ceske Budejovice Ceske Budejovice the Czech Republic

**Keywords:** Clinical practice, cultural competence, cultural diversity, nurses, nursing education, patient care

## Abstract

**Aim:**

This study aimed to compare the level of cultural competence among nurses working in clinical practice in Slovakia and the Czech Republic.

**Background:**

Demographic changes have greatly affected the health sector in Slovakia and the Czech Republic. By identifying the level of nurses’ cultural competence, many of the complications encountered in caring for patients from different cultures can be avoided. However, few studies have explored the cultural competence of nurses in clinical practice in these countries.

**Method:**

This study was cross‐sectional, descriptive, and comparative. It followed the STROBE checklist and used the Cultural Competence Assessment Tool questionnaire to collect data. Descriptive and inferential statistical tests were utilized for data analysis, using SASD 1.5.8 and IBM SPSS Statistics version 28.0.0.

**Results:**

The sample comprised 424 nurses, with 202 from the Czech Republic and 222 from Slovakia, primarily female. Most nurses in both countries have not received cultural diversity training. Nevertheless, nurses in both countries indicate the necessity of conducting a cultural impact assessment of patients’ health. Cultural diversity training significantly increases the level of cultural competence in nurses.

**Discussion:**

Lower cultural competence scores can negatively impact nursing care for patients from different cultures, leading to additional cultural challenges.

**Implications for nursing and health policy:**

The findings highlight the need for enhanced cultural competence among nurses. Nurses need to learn and utilize cultural information to help maximize healthcare for patients from different cultures. By providing nurses with cultural knowledge and skills, they will be able to deliver more effective and culturally competent care to patients from varied cultural backgrounds.

## INTRODUCTION

Culturally competent nursing practice is crucial due to increased cultural diversity in societies and human migration (Sharifi et al., 2019). However, culturally congruent care cannot exist without cultural competence as it is an essential feature of high‐quality healthcare for patients from different cultures (McGregor et al., 2019). The nursing care processes currently in place may not accommodate the culturally specific requirements of patients from diverse backgrounds. This gap in service quality could create unresolved health problems for patients, resulting in further illnesses that could have been prevented had quality, culturally sensitive care been provided to patients from different cultures (Omenka et al., 2020; Williams et al., 2019). Therefore, it is imperative to conduct further research to evaluate the level of cultural competence among nurses and pinpoint potential obstacles to deliver culturally competent care. Societal demographic changes pose a challenge not just to the Member States of the European Union (EU) but also to their healthcare systems. For instance, it is apparent that healthcare professionals, including nurses, often encounter difficulties owing to cultural disparities with patients. Nurses express concern regarding potential cultural boundary crossings owing to insufficient knowledge of patients' cultures. Nurses’ lack of expertise and understanding of the unique cultural impacts on health are of particular concern. The broad spectrum of cultures represented in society exacerbates communication breakdowns in the healthcare, leading to feelings of uncertainty, discomfort, and confusion when attempting to provide culturally appropriate care (Červený et al., 2022).

According to the 2022 Eurostat report, as of January 1, 2021, 23.7 million people were living in the territory of the European Union who did not have citizenship from an EU country. Overall, this number represented 5.3% of the EU population. According to the Institute of Health Information and Statistics of the Czech Republic, in 2021, inpatient healthcare was provided on its territory for 148,500 patients of other nationalities, which is expected to grow. Healthcare was provided to patients from Slovakia (25%), Ukraine (19%), Russia (6%), Vietnam (6%), and Germany (5%). The number of foreigners in the Slovak Republic has increased almost eight‐fold since joining the EU. Healthcare data from Slovakia are not published, but as of December 31, 2021, 167,519 foreigners were living in Slovakia (ÚHCP P PZ, 2021). According to the systematic review's findings, marginalized groups often receive culturally incompetent healthcare of lower quality. Additionally, these communities reportedly face more frequent diagnostic delays, resulting in lower patient safety due to medication errors (Cheraghi‐Sohi et al., 2020).

### What is cultural competence, and why is it essential for current practice?

Cultural competence does not have a clear and uniform definition (Antón‐Solanas et al., 2021; Curtis et al., 2019; Ślusarska et al., 2022). Cultural competence is the ability to improve the healthcare system by integrating culture into caring for patients from different cultures (Lau & Rodgers, 2021). However, the concept of cultural competence in healthcare can be a topic of debate. Currently, there is consideration that, to achieve equality in healthcare service provision, cultural safety is required over cultural competence (Curtis et al., 2019). This is because cultural competence is a broad term that covers various components such as cultural awareness, humility, sensitivity, and respect (Antón‐Solanas et al., 2021). Cultural competence requires a systematic understanding of cultural and social factors. Although there are other definitions and characterizations of cultural competence in the literature, the critical feature is that it provides a mechanism for improving healthcare provision (Kula et al., 2021). It can improve the quality of care provided and, most importantly, reduce health disparities. Cultural safety emphasizes the recognition and understanding of power disparities. It implies that safe and high‐quality treatment can be achieved if healthcare professionals are aware of power disparities and evaluate the impact of their cultural background, preconceptions, stereotypes, and other factors on clinical communication with patients (Curtis et al., 2019).

Evidence suggests that unconscious prejudices of healthcare providers toward people of different cultures or ethnicities and the tendency to stereotype patients can have detrimental consequences for patient care. These consequences can include poor communication between patients and providers, lack of coordination between healthcare professionals, lack of patient trust in the healthcare system, and reduced patient satisfaction with the care they receive (Červený et al., 2022; Schouler‐Ocak et al., 2021; Sim et al., 2021). Health workers, including nurses, have reported problems and cultural challenges in providing healthcare to patients from different cultures (Červený et al., 2019; Gutysz‐Wojnicka, 2022; Ślusarska et al., 2022). For this reason, it is important to explore the cultural competence of nurses in clinical practice.

### Aim of the study

The study aimed to compare the level of cultural competence of nurses working in clinical practice in the Czech Republic and Slovakia.

## METHODS

### Research design

This cross‐sectional, descriptive, and comparative study used an online survey method. The research team chose the design to objectively measure and compare the exposures and outcomes of the phenomenon (Wang & Cheng, 2022) resulting from the current clinical practice of nurses. To report this study, the researchers adhered to the STROBE checklist (von Elm et al., 2008).

### Settings and participants

The research sample consisted of nurses working in clinical practice in Slovakia and the Czech Republic. Nurses in the Czech Republic and Slovakia receive comparable professional education at the pre‐ and graduation levels. Consequently, these two countries were selected for the study and subsequent comparison of results. The sample sets were created using a simple random sampling method. By implementing Slovin's method, the research team calculated that the sample size should be 383 to achieve a 95% confidence level and a 5% margin of error. Participant selection criteria included: (1) nurses currently working in any setting (such as hospitals, schools, clinics); (2) working full‐time or part‐time; (3) providing direct nursing care to patients; and (4) consented to participate in this study.

### Data collection and measurement

The study was conducted between early June and November 2022. In Slovakia, data collection was conducted through the web portal of the Slovak Chamber of Nurses and Midwives (SkSaPa). Nurses were invited to participate in the research through an email invitation sent to their registered email addresses in SkSaPa. In the Czech Republic, data collection took place in Czech hospitals. We contacted the nurse directors of hospitals, requesting the questionnaire to be made available to nurses through the hospital's information system. From a legislative perspective, Slovak nurses practicing in clinical settings must be registered with the relevant healthcare chamber (Slovak Act No. 578/2004). In contrast, Czech nurses are not required to register (Czech Act No. 96/2004 and its amendment as of September 1, 2017). Therefore, we adopted a specific approach to data collection by directly approaching hospitals. This ensured that respondents from clinical practice were included in our study. We collected data using a standardized questionnaire, i.e., the Cultural Competence Assessment Instrument developed by Schim et al. (2003). At the end of the questionnaire, we collected demographic data (age, education, job position, completion of a course focused on transcultural nursing, etc.) from the participants, which was used to detect correlation relationships.

The cultural competence assessment instrument (CCA) measures cultural competence in nurses in clinical practice. It integrates two subscales: (1) the Cultural Awareness and Sensitivity (CAS) scale and (2) the Culturally Competent Behavior (CCB) scale. The CAS subscale consists of questions 6–16, where participants had an opportunity to answer questions using a 7‐point Likert scale ranging from 1 (strongly agree) to 7 (completely disagree). The CCB subscale consists of questions 17–30, where participants could express their views using a 7‐Likert point scale ranging from 1 (always) to 7 (never) (Červeny et al. 2020).

The interpretation of the results derived from the CCA instrument suggests that the obtained scores may range from 25 to 125, with higher scores indicating a higher level of cultural competence. The CAS subscale's scoring system indicates a higher average reflects greater cultural awareness and sensitivity (Osmancevic et al., 2023). The CCB subscale scoring system indicates that a higher mean score signifies greater competence in exhibiting cultural competence and behaviors. The CCA is not available in Czech, so it was translated into Czech, where (1) two independent translators translated the questionnaire from English into Czech; (2) the Czech version was reverse‐translated back into English for comparison; and (3) the English language versions of CCA were reviewed for consensus. Consent to use CCA was obtained from the authors of the questionnaire. The Cronbach alpha CAS‐CZ version was 0.627, and the CCB‐CZ version was 0.921, reflecting strong item coherence. The Cronbach alpha subscale of the CAS‐SK version was 0.693, and the subscale of the CCB‐SK version was 0.919, which also reflects robust item coherence.

### Ethical considerations

Each participant in this study was informed about the purpose of the research. Participation was voluntary and anonymous, and the results were used for research purposes only. No identifying data about the participants were collected. By completing the questionnaire, participants agreed to participate in the research. The research was approved by the Ethics Committee of the University of South Bohemia in Ceske Budejovice, Faculty of Health and Social Sciences (October 11, 2020).

### Data analysis

Descriptive statistics (percentages, mean, mode, and standard deviation) were used to analyze the data. In addition, statistical tests such as Pearson Chi‐square test, independence test, Pearson coefficient contingency, Ćuprov coefficient, Cramer, Wallis, and Spearman coefficient were used. The statistical significance was set at *p* < 0.05, *p* < 0.001, and *p* < 0.001. Statistical analysis was performed in SASD 1.5.8 and IBM SPSS Statistics version 28.0.0 (IBM Corp., Armonk, NY, USA).

## RESULTS

### Participant characteristics

Four hundred and twenty‐four (n = 424) nurses participated in the study, of which 202 were from the Czech Republic (CZ group) and 222 were from Slovakia (SK group). The average age in the CZ group was 41.40 years; in the SK group, it was 43.51 years. Most participants were female (402), with 192 in the CZ group and 210 in the SK group, respectively.

The level of education varied; participants had education from secondary medical school to a Ph.D. in nursing. The average length of experience in clinical practice was 22 years in the CZ group and 21 years in the SK group. Most participants had not received training in cultural diversity in either the Czech Republic or Slovakia (Table 1).

**TABLE 1:** Characteristics of participants.

	Group SK	Group CZ
Characteristics	*n* = 222 (100%)	*n *= 202 (100%)
Age		
<29	39 (17.6)	28 (13.9)
30–39	39 (17.6)	48 (23.8)
40–49	74 (33.3)	62 (30.7)
50–59	57 (25.7)	52 (25.7)
60<	13 (5.9)	12 (5.9)
Gender		
Female	210 (94.6)	192 (95.0)
Male	12 (5.4)	10 (5.0)
Education level		
Secondary Schools of Nursing	34 (15.3)	54 (26.7)
Nursing diploma—higher	35 (15.8)	44 (21.8)
BSc degree	66 (29.7)	49 (24.3)
MSc degree	75 (33.8)	52 (25.7)
PhD degree	12 (5.4)	3 (1.5)
Years of clinical practice		
1−2	8 (3.6)	9 (4.5)
3−5	26 (11.7)	19 (9.4)
6−10	37 (16.7)	22 (10.9
11−20	37(16.7)	47 (23.3)
21<	113 (50.9)	105 (52.0)
M ± SD	56.30 ± 43.91	57.76 ± 43.18
Training in cultural diversity		
Yes	32 (14.4)	64 (31.7)
No	190 (85.6)	38 (68.3)
Total	222 (100)	202 (100)

### CAS among nurses in clinical practice

Table 2 (Section A) shows the results of the assessment of cultural awareness among nurses. The results show that the CZ group had a significantly higher average score than the SK group. Nurses in both groups had a similar assessment of the importance of ethnicity in determining the culture of a person (Q6). The SK group had significantly higher average scores (4.21 ± 1.69) than the CZ group (3.28 ± 1.36) when reporting that people with a common cultural background think and act alike. While the CZ group had a higher average score (2.77 ± 1.29) than the SK group (2.30 ± 1.26), both groups agree on the importance of assessing cultural aspects in each patient/person (Q9). When assessing the language barrier as the sole cultural challenge faced by immigrants, both groups acknowledged that language is not the only cultural challenge. However, the Czech group displayed a tendency to strongly disagree with this statement (Q13).

**TABLE 2:** Self‐reported cultural competence awareness and CCB among nurses.

	Mean ± SD	
A: Cultural Competence Awareness and Sensitivity (CAS)	Group SK (*n* = 222)	Group CZ (*n* = 206)
*Q6*. Race is the most important factor in determining a person's culture.	4.05 ± 1.87	4.13 ± 1.58
*Q7*. People with a common cultural background think and act alike.	4.21 ± 1.69	3.28 ± 1.36
*Q8*. Many aspects of culture influence health and healthcare.	2.28 ± 1.06	2.48 ± 1.13
*Q9*. Aspects of cultural diversity need to be assessed for each individual, group, and organization.	2.30 ± 1.26	2.77 ± 1.29
*Q10*. If I know about a person's culture, I do not need to assess their personal preferences for health services.	5.00 ± 1.72	4.77 ± 1.57
*Q11*. Spiritual and religious beliefs are important aspects of many cultural groups.	2.31 ± 1.19	2.50 ± 1.04
*Q12*. Individual people may identify with more than one cultural group.	2.95 ± 1.48	2.87 ± 1.26
*Q13*. Language barriers are the only difficulties for recent immigrants.	4.23 ± 2.01	4.88 ± 1.89
*Q14*. I believe everyone should be treated with respect regardless of their cultural heritage.	1.62 ± 0.78	2.09 ± 1.24
*Q15*. I understand that people from different cultures may define the concept of "healthcare" in different ways.	1.90 ± 0.83	2.19 ± 1.02
*Q16*. I think knowing about different cultural groups helps direct my work with individuals, families, groups, and organizations.	2.25 ± 1.18	2.49 ± 1.10
Total	1.75 ± 2.94	3.13 ± 1.65

	Mean ± SD	
B: Cultural Competence Behavior (CCB)	Group SK (*n* = 222)	Group CZ (*n* = 206)
*Q17*. I include a cultural assessment when I do individual or organizational evaluations.	3.39 ± 1.92	4.44 ± 1.83
*Q18*. I seek information on cultural needs when I encounter new people at my work or school.	4.52 ± 1.91	5.01 ± 1.68
*Q19*. I have resource books and other materials to help me learn about people from different cultures.	4.60 ± 1.73	5.34 ± 1.83
*Q20*. I use a variety of sources to learn about the cultural heritage of other people.	4.46 ± 1.75	4.54 ± 1.74
*Q21*. I ask people to explain or describe their health and illnesses.	4.17 ± 2.00	5.59 ± 1.65
*Q22*. I ask people to tell me about their health services expectations.	4.22 ± 1.95	5.06 ± 1.70
*Q23*. I avoid using generalizations to stereotype groups of people.	3.97 ± 2.07	3.96 ± 1.95
*Q24* I recognize potential barriers to service that might be encountered by different people.	4.13 ± 1.77	3.74 ± 1.57
*Q25*. I remove obstacles for people of different cultures when I identify barriers to services.	4.05 ± 1.83	4.57 ± 1.63
*Q26*. I remove obstacles for people of different cultures when people identify barriers to me.	4.10 ± 1.91	4.80 ± 1.70
*Q27*. I welcome client feedback about how I relate to people from different cultures.	4.02 ± 1. 87	4.47 ± 1.93
*Q28*. I find ways to adapt my services to individual and group cultural preferences.	4.15 ± 1. 88	4.42 ± 1.79
*Q29*. I document cultural assessments when I provide direct client services.	5.45 ± 1.76	6.05 ± 1.49
*Q30*. I document the adaptations I make with clients when I provide direct client services.	5.19 ± 1.98	5.87 ± 1.62
Total	4.31 ± 1.94	4.85 ± 1.85

*Note*. SD: standard deviation.

### CCB of nurses in the Czech Republic and Slovakia

Table 2 (Section B) shows the assessment of cultural behavior between the SK and CZ groups. The overall average score was 4.31 ± 1.94 in group SK and 4.85 ± 1.85 in group CZ. The results show that the CZ group had a higher average score than the SK group, indicating that nurses in the CZ group were likely to pay more attention to the cultural needs of patients. The lowest values in the SK group were on Q17 (“I include cultural assessment when I do individual or organizational evaluations.”) and in group CZ on Q24 (“I recognize potential barriers to service that might be encountered by different people.”). The highest average scores were similar in the SK and CZ groups, indicating that both groups of nurses agree that documenting cultural information in the provision of care is essential.

### Cultural competence in Slovak and Czech nursing groups

We found a strong correlation between the CAS, CCB, and the education of nurses in the SK group. In the CZ group, this significant relationship existed only in the CAS. Completing training focused on cultural diversity also showed a significant relationship between the SK group on the CCB. However, completing cultural diversity training in the CZ group only affected CCB results.

## DISCUSSION

Cultural competence is a fundamental aspect of providing culturally appropriate care. This investigation assessed, analyzed, and compared the cultural competence of nurses working in Slovakia and the Czech Republic. The findings provide valuable perspectives into the factors affecting cultural competence among nurses in these two nations. Since the Czech Republic and Slovakia share a rich cultural and social history (Tóth et al., 2023), the results hold significance both nationally and internationally. The results reveal that age had a significant impact solely on the CCB (*p* < 0.015) in group SK when compared with group CZ. This finding implies that age potentially plays a crucial role in shaping cultural competence, though further research is needed to identify and comprehend its underlying reasons. In addition, the length of time in clinical practice demonstrated an influence solely within the SK group on CCB. Similar conclusions were drawn by Cai et al. (2021) from China. They suggest that nurses obtain essential skills in treating patients from various cultures through practice. When considering other nations in Northern Europe (Denmark, Finland, Iceland, Norway, and Sweden), Western Europe (Belgium, Ireland, the Netherlands, and the United Kingdom), and Southern Europe (Italy, Spain, and others), nurses hailing from these countries displayed higher scores of willingness to offer diverse healthcare to patients from diverse cultures when compared with Eastern European countries, as statistical analysis reveals (Červený et al., 2019). Regardless of findings showing that cultural competence has been neglected in Slovakia and the Czech Republic, a comparable pattern has also been detected in China. The outcomes of the study suggest that nursing education facilities should provide cultural competence training to nurses and customize training programs to meet the unique requirements of different nursing groups, consequently enhancing the effectiveness of training (Cai et al., 2021).

It is worth mentioning that the nurse's professional position in the CZ group had a statistical impact on the CAS and CCB, whereas, in the SK group, this relationship was present only for the CCB. The findings regarding the job position are interesting. In the SK group, nurses with lower professional positions have higher CCB scores. However, the head nurses in the CZ group had higher CAS and CCB scores. We can explain this as head nurses are closest to patients on a daily basis; however, they already perform the role of nursing care manager in the ward. This finding is consistent with the results of Wang et al. (2022), where the authors found that higher job positions were associated with higher cultural competence. Their reasoning is like ours in that nurses in senior management positions are more sensitive to cultural needs in clinical practice. Cultural differences can lead to misunderstandings, reduced safety, increased health risks, as well as discrimination and racial/ethnic inequalities in the provision of healthcare (Qureshi et al., 2022; Williams et al., 2019).

This study found that the level of nursing education had a significant positive impact on cultural competence. In the SK group, the higher level of education impacted both CAS and CCB, while in the CZ group, education only affected the CAS (Table 3). This finding is consistent with previous findings (Cai et al., 2021; Červený et al., 2020; Marja & Suvi, 2021; Osmancevic et al., 2023). Finally, Cicolini et al. (2015) noted that university‐educated nurses in Italy were exposed to cultural diversity training during their studies, requiring culturally appropriate understanding and behaviors. However, the results from Poland and Lithuania are noteworthy, where the authors found that nurses with lower levels of education had better cultural competence scores, NCCS‐CK cultural knowledge (*p* < 0.01), and NCCS‐CS cultural skills (*p* < 0.01) subscales, as well as in general—NCCS (*p* < 0.01). Nursing education institutions or hospitals should increase cultural competence training since the cultural competence of healthcare professionals can improve the overall experience and satisfaction of patients during their hospitalization. Cultural competence emphasizes mindful, congruent, and patient‐oriented care (Carey, 2011; Weech‐Maldonado et al., 2012). The analysis of the findings showed that Slovak nurses who completed cultural diversity training had higher scores on CCB. These scores were higher only for CCB in Czech nurses. These results suggest that trained nurses in cultural diversity in the Czech Republic and Slovakia exhibit CCB in clinical practice. These results align with previous studies conducted in Austria (Osmancevic et al., 2023), Italy (Cicolini et al., 2015), and Poland (Majda et al., 2021).

**TABLE 3:** Relationship between variables and cultural competence.

	Group SK (*n* = 222)		Group CZ (*n* = 206)	
Characteristics	CAS Mean ± SD	CCB Mean ± SD	CAS Mean ± SD;	CCB Mean ± SD
Gender				
Female	2.73 ± 0.60	4.00 ± 1.20	2.93 ± 0.64	4.57 ± 1.12
Male	2.59 ± 0.72	3.90 ± 1.37	2.77 ± 0.48	4.87 ± 0.98
Total	2.73 ± 0.62	3.99 ± 1.20	2.92 ± 0.63	4.58 ± 1.11
Significance	*p* = 0.618	*p* = 0.867	*p* = 0.411	*p* = 0.467
Age (years)	43.51 ± 11.24		43.41 ± 11.02	
Significance	*p* = 0.517	*p* < 0.015^*^	*p* = 0.416	*p* = 0.403
Current role				
Nurse	2.71 ± 0.62	4.14 ± 1.19	2.91 ± 0.67	4.76 ± 1.02
Head nurse	2.86 ± 0.36	3.87 ± 0.98	3.12 ± 0.45	4.89 ± 0.79
Chief nurse of the department	2.71 ± 0.67	3.51 ± 1.21	2.65 ± 0.53	4.00 ± 1.24
Total	2.73 ± 0.62	3.99 ± 1.20	2.92 ± 0.63	4.58 ± 1.11
Signification	*p* = 0.138	*p* < 0.042^*^	*p* < 0.002^**^	*p* < 0.004^**^
Years of experience in clinical practice	20.71 ± 12.76		31.14 ± 138.34	
Significance	*p* = 0.395	*p* < 0.033^*^	*p* = 0.212	*p* = 0.604
Educational level				
Secondary School of Nursing	2.98 ± 0.66	4.49 ± 1.28	3.22 ± 0.58	4.75 ± 1.14
Nursing diploma	2.89 ± 0.67	4.13 ± 1.20	3.03 ± 0.59	4.53 ± 1.03
BSc in Nursing	2.70 ± 0.55	4.08 ± 1.12	2.76 ± 0.68	4.62 ± 1.11
MSc in Nursing	2.60 ± 0.58	3.80 ± 1.10	2.70 ± 0.52	4.39 ± 1.17
Ph.D. in Nursing	2.41 ± 0.60	2.98 ± 1.24	2.52 ± 0.84	4.86 ± 0.40
Total	2.73 ± 0.62	3.99 ± 1.20	2.92 ± 0.63	4.58 ± 1.11
Significance	*p* < 0.007^**^	*p* < 0.008^**^	*p* < 0.000^***^	*p* = 0.545
Prior diversity training				
Yes	2.70 ± 0.61	3.39 ± 1.06	2.84 ± 0.62	4.27 ± 1.20
No	2.72 ± 0.61	4.11 ± 1.19	2.96 ± 0.63	4.73 ± 1.03
Total	2.73 ± 0.62	3.99 ± 1.20	2.92 ± 0.63	4.58 ± 1.11
Significance	*p* < 0.802	*p* < 0.002^**^	*p* = 0.265	*p* < 0.016^*^

* Correlation is significant at *p* < 0.05.

** Correlation is significant at *p* < 0.01.

*** Correlation is significant at *p* < 0.001.

### Strength and limitations

This study has several weaknesses and strengths. The size of the research population limits the generalization of results to the entire nursing population in the Czech Republic and Slovakia. Furthermore, the authors did not collect specific data on the educational training participants had received on cultural diversity training. The strength of this study is that no previous studies were identified in the Czech literature that have explored the cultural competence of nurses working in clinical practice, and therefore, this study's findings provide an important contribution to addressing this gap in knowledge.

## CONCLUSION AND RECOMMENDATIONS

This study identifies the factors influencing the cultural competence of nurses in clinical practice in the Czech Republic and Slovakia. Czech nurses score higher on average in cultural competence than nurses from Slovakia. However, the results indicate that nurses in both groups were aware of the significance of offering culturally sensitive care to patients from diverse cultures. Additionally, statistical analysis showed that cultural competence was significantly associated with variables such as age, job position, duration of clinical practice, education, and completion of cultural diversity training. Recognizing and addressing the factors that impact cultural competence in nursing practice is imperative for delivering quality, patient‐centered care to individuals from varied cultural backgrounds. Healthcare systems can overcome any existing gaps by determining the level of cultural competence of nurses in clinical practice and adapting their educational and training programs accordingly. Further investigation is necessary to analyze the factors and aspects that impact the development of cultural competence in clinical practice for nurses.

## IMPLICATIONS FOR NURSING PRACTICE AND HEALTHCARE POLICY

The findings of this study carry significant implications for enhancing culturally competent care in healthcare, not only within specific countries but also in an international context. To ensure high‐quality healthcare for patients from diverse cultures and enhance their contentment with the rendered services, healthcare institutions and nursing education policies should proactively address the difficulties related to cultural diversity. Unresolved cultural challenges, including inadequate comprehension of patients' religious and cultural dietary requirements, health support, health beliefs, and use of alternative healing practices due to cultural disparities, may lead to further health complications, placing undue pressure on the healthcare system. The entire health system is affected by demographic and cultural changes in society. Therefore, nurses need to recognize and analyze the impact of culture on the entire nursing care process to mitigate potential disparities, decrease discrimination, and enhance patient outcomes. Furthermore, including cultural competence training in undergraduate nursing education equips future nurses to effectively address the healthcare needs of patients from diverse cultural backgrounds. These results recommend that employers provide nurses with educational activities, courses, simulation training, or other cultural diversity training activities. As not every nurse will encounter cultural competence training during their initial nursing education program healthcare institution managers should provide educational training to enhance cultural competence, improving care for patients from diverse cultures and enhancing the quality of healthcare delivery. These implications extend beyond the Czech Republic and Slovakia borders, as cultural diversity is a global phenomenon.

## AUTHORSHIP CONTRIBUTIONS

*Study design*: MC, VT. *Data collection*: MC, VT. *Data management*: MC, VT. *Data analysis*: MC, VT. *Study supervision*: MC, VT. *Manuscript writing*: MC, VT.

## CONFLICT OF INTEREST STATEMENT

No conflicts of interest are declared by the authors.

## ETHICAL CONSIDERATIONS

Each participant in this study was informed about the purpose of the research. Participation was voluntary and anonymous, and the results were used for research purposes only. No identifying data about the participants were collected. By completing the questionnaire, participants agreed to participate in the research. The research was approved on November 19, 2020, by the Ethics Committee of the Faculty of Health and Social Sciences, University of South Bohemia in České Budějovice.
